# Innovative prognostication: a novel nomogram for post-interventional aneurysmal subarachnoid hemorrhage patients

**DOI:** 10.3389/fneur.2024.1410735

**Published:** 2024-08-19

**Authors:** Qinyu Guo, Hongyi Chen, Shirong Lin, Zheng Gong, Zhiwei Song, Feng Chen

**Affiliations:** ^1^Department of Emergency, Fujian Provincial Hospital, Fujian, Fuzhou, China; ^2^Shengli Clinical Medical College, Fujian Medical University, Fujian, Fuzhou, China; ^3^Fujian Provincial Institute of Emergency Medicine, Fujian Provincial Hospital, Fujian, Fuzhou, China; ^4^Fuzhou University Affiliated Provincial Hospital, Fujian, Fuzhou, China; ^5^Fujian Emergency Medical Center, Fujian Provincial Hospital, Fujian, Fuzhou, China; ^6^Fujian Provincial Key Laboratory of Emergency Medicine, Fujian Provincial Hospital, Fujian, Fuzhou, China

**Keywords:** aneurysmal subarachnoid hemorrhage, interventional embolization, prediction model, functional outcomes, validation

## Abstract

**Background and purpose:**

Spontaneous aneurysmal subarachnoid hemorrhage (aSAH) is a common acute cerebrovascular disease characterized by severe illness, high mortality, and potential cognitive and motor impairments. We carried out a retrospective study at Fujian Provincial Hospital to establish and validate a model for forecasting functional outcomes at 6 months in aSAH patients who underwent interventional embolization.

**Methods:**

386 aSAH patients who underwent interventional embolization between May 2012 and April 2022 were included in the study. We established a logistic regression model based on independent risk factors associated with 6-month adverse outcomes (modified Rankin Scale Score ≥ 3, mRS). We evaluated the model’s performance based on its discrimination, calibration, clinical applicability, and generalization ability. Finally, the study-derived prediction model was also compared with other aSAH prognostic scales and the model’s itself constituent variables to assess their respective predictive efficacy.

**Results:**

The predictors considered in our study were age, the World Federation of Neurosurgical Societies (WFNS) grade of IV-V, mFisher score of 3–4, secondary cerebral infarction, and first leukocyte counts on admission. Our model demonstrated excellent discrimination in both the modeling and validation cohorts, with an area under the curve of 0.914 (*p* < 0.001, 95%CI = 0.873–0.956) and 0.947 (*p* < 0.001, 95%CI = 0.907–0.987), respectively. Additionally, the model also exhibited good calibration (Hosmer-Lemeshow goodness-of-fit test: X^2^ = 9.176, *p* = 0.328). The clinical decision curve analysis and clinical impact curve showed favorable clinical applicability. In comparison to other prediction models and variables, our model displayed superior predictive performance.

**Conclusion:**

The new prediction nomogram has the capability to forecast the unfavorable outcomes at 6 months after intervention in patients with aSAH.

## Introduction

Spontaneous Aneurysmal Subarachnoid Hemorrhage (aSAH) is a common and acute cerebrovascular condition that is often seen in neurological emergency departments. It is characterized by its profound severity and is connected with high mortality rates and enduring disability. As a result, it can place substantial socio-economic burdens on both society and afflicted families.

Several scoring systems or prediction models have been established to evaluate prognosis after aSAH, including the aSAH prognostic prediction model developed from the Subarachnoid Hemorrhage International Trialists (SAHIT) (1) multinational cohort study, the Functional Recovery Expected after Subarachnoid Hemorrhage (FRESH) (2), and the SAFIRE grading scale developed by van Donkelaar et al. (3). It is worth noting that these scoring systems are typically designed to apply to a broad spectrum of subarachnoid hemorrhage cases. Currently, surgical interventions are crucial in the management of aSAH patients. Microsurgical aneurysm clipping and endovascular interventional embolization not only prevent rebleeding, but also potentially mitigate severe complications such as cerebral herniation, hydrocephalus, and vasospasm. It is noteworthy that different treatments may exert varying influences on patient prognosis. Therefore, the refinement and stratification of study populations enable the attainment of more precise predictive results.

Nevertheless, most existing studies on prediction models in aSAH patients demonstrate notable deficiencies in terms of comprehensive and practical predictive methods. For instance, they frequently fall short in the collection of relevant biomarkers from both blood and cerebrospinal fluid during prognostic investigations, thereby neglecting the potentially pivotal role of neurobiology in anticipating outcomes after aSAH (4, 5). Furthermore, some prediction models primarily gather data related to preoperative patient conditions while disregarding critical elements such as intraoperative rebleeding, acute thrombosis formation, delayed cerebral ischemia, and others (6). Additionally, certain studies have focused on populations with unique living environments, lifestyle habits, and genetic backgrounds, limiting the predictive value of their models for the broader Chinese population (7). To establish accurate and reliable prediction models, further exploration is needed.

To overcome these limitations, we sought to develop a model to predict the 6-month prognosis of patients with aSAH undergoing endovascular aneurysm embolization, with the goal of helping clinicians to identify high-risk and to provide a more proactive treatment strategy.

## Materials and methods

### Patient selection

Our study collected data at an early stage on all patients with spontaneous aSAH who underwent interventional embolization at our hospital and branch hospitals between May 2012 and April 2022. The cases collected at our hospital constituted the modeling cohort, while those collected at our branch hospital were used as validation cohort.

Inclusion criteria: (1) age > 18 years and the first occurrence of aSAH; (2) onset of symptoms within 72 h and confirmed the etiology of SAH as intracranial aneurysm rupture through head Computed Tomography (CT) and/or whole-brain Digital Subtraction Angiography (DSA) examinations; and (3) underwent endovascular embolization treatment.

Exclusion criteria: (1) non-aneurysmal causes of subarachnoid hemorrhage; (2) coexistence of other cerebrovascular diseases such as arteriovenous malformation, arteriovenous fistula, moyamoya disease; (3) patients with severe systemic diseases before the onset of the condition, such as hematological disorders, immune system disorders, recent history of central nervous system infection or other infectious diseases, severe cardiopulmonary, hepatic, and renal insufficiencies; (4) pregnant and postpartum women; and (5) patients with incomplete or lost follow-up data during hospitalization.

The Fujian Provincial Hospital Ethics Committee authorized this retrospective observational study (K2021-07-044).

### Data collection

We collected the patients’ basic status information during hospital admission, which included demographic and clinical characteristics (8, 9), time of onset, laboratory examination and medical histories, such as diabetes and hypertension. The study scope also covered clinical assessments and In-hospital care, including clinical scores that evaluate aSAH severity (10–14), characteristics of the aneurysm (4), timing of interventional surgery, cerebrospinal fluid drainage (15), post-rupture complications, duration of mechanical ventilation, and length of hospital stay (16). Post-rupture complications included secondary cerebral infarction (17, 18), rebleeding (19, 20), pulmonary infection (21, 22), hydrocephalus, seizures (23, 24), cerebral herniation and cardiac events (25). In particular, secondary cerebral infarction refers to new cerebral ischemic lesions occurring after spontaneous subarachnoid hemorrhage due to various possible reasons such as vasospasm, thrombus formation, delayed cerebral ischemia, etc.

### Prognostic outcome measures

Clinical follow-up of patients was conducted at 6 months postoperatively through outpatient visits and telephone consultations. The prognosis was evaluated using the modified Rankin Scale (mRS), where a score of 0–2 indicated a positive prognosis and a score of 3–6 indicated an unfavorable prognosis.

### Statistical analysis

The statistical analyses were executed using the software SPSS 25.0 and R 4.2.2. Quantitative data that conformed to normal distribution were expressed as mean ± standard deviation (SD), while non-normally distributed quantitative data were presented as median and interquartile range (IQR). Counts and percentages [cases (%)] were used for categorical data. Student’s *t*-test or Mann–Whitney U test was employed for the comparison of quantitative variables, as appropriate, and the chi-square test or Fisher’s exact test was utilized for comparing categorical variables. Multicollinearity was assessed using the tolerance and variance inflation factor (VIF). To identify independent risk factors associated with unfavorable outcomes at 6 months, only variables from the univariate analysis that had a significance level of *p* ≤ 0.01 were included in the multivariate logistic regression analysis using a backward stepwise regression. The associations were conveyed by using odds ratios (ORs) along with their respective 95% confidence intervals (CIs).

The prediction model was crafted utilizing the rms package in the R language. The evaluation of the prediction model’s performance involved the utilization of the following validation methods. The plotting and calculation of ROC curves and AUC for both the modeling and validation cohorts were conducted using the pROC package. The rms package was used to plot and calculate the calibration curves and brier scores for the two cohorts, while the ResourceSelection package was utilized to conduct the Hosmer-Lemeshow test. The rmda package was employed for generating clinical decision curves and clinical impact curves for both cohorts, and the caret package was used to run the bootstrap self-sampling method. Finally, the riskRegression package and pROC package were utilized to compare the ROC curves of the prediction model with those of other aSAH prognostic rating scales and the component variables of the model.

## Results

### Patient characteristics

Our study collected data at an early stage on all patients with spontaneous aSAH who underwent interventional embolization at our hospital and branch hospitals between May 2012 and April 2022, a total of 446 cases. Of these, 60 patients were excluded, including one patient under 18 years old, five readmitted due to recurrent episodes, two cases where embolization was terminated during surgery, eight cases where no aneurysm was found on angiography, 19 cases with symptom onset beyond 72 h, and 25 cases lost to follow-up. Thus, the final 386 cases were included in the study. Of the 258 patients who underwent interventional embolization at our hospital from May 2012 to April 2022, they were included as the modeling cohort. The number of such patients at our branch hospital from April 2017 to April 2022 was 128, and they were included as the validation cohort. Both independent sets were further categorized into groups denoting either favorable or unfavorable outcomes based on the functional recovery at 6 months after onset.

### Variable selection

Tables 1–3 show the modeling cohort’s univariate analysis results. Adverse prognosis in aSAH patients with interventional embolization is related with age (*p* < 0.001), respiratory rate (*p* < 0.001), GCS (Glasgow Coma Scale) score (*p* < 0.001), WFNS (World Federation of Neurological Surgeons) grade (*p* < 0.001), mFisher grade (*p* < 0.001), Hunt-Hess grade (*p* < 0.001), first leukocyte counts on admission (*p* < 0.001), neutrophil count (*p* < 0.001), lymphocyte count (*p* = 0.002), monocyte count (*p* = 0.003), D-dimer level (*p* < 0.001), duration of mechanical ventilation (*p* < 0.001), number of cerebrospinal fluid drainage modalities (*p* < 0.001), secondary brain infarction (*p* < 0.001), pulmonary infection (*p* < 0.001), hydrocephalus (*p* = 0.001), brain herniation (*p* < 0.001), and cardiac events (*p* < 0.001).

**Table 1:** Baseline information of the aSAH study cohort.

Variables	Modeling cohort (*N* = 258)				Validation cohort (*N* = 128)	
	Favorable outcome (*n* = 182)	Unfavorable outcome (*n* = 76)	Value	*p*	Favorable outcome (*n* = 95)	Unfavorable outcome (*n* = 33)
**Demographic characteristics**						
Male, *n* (%)	102 (56)	44 (57.9)	0.075^b^	0.785	58 (61.1)	17 (51.5)
Age, mean ± SD, (year)	53.3 ± 10.7	61.0 ± 12.2	−4.795^a^	**<0.001**	55.7 ± 10.1	59.1 ± 12.3
**Medical history**						
Hypertension, *n* (%)	98 (53.8)	43 (56.6)	0.162^b^	0.688	51 (53.7)	16 (48.5)
Diabetes, *n* (%)	17 (9.3)	6 (7.9)	0.138^b^	0.710	7 (7.4)	3 (9.1)
Smoking, *n* (%)	44 (24.2)	13 (17.1)	1.557^b^	0.212	22 (23.2)	6 (18.2)
Drinking, *n* (%)	32 (17.6)	10 (13.2)	0.770^b^	0.380	10 (10.5)	3 (9.1)
**Admission condition**						
temperature, median[IQR], (°C)	36.6 (36.5, 36.8)	36.7 (36.5, 36.9)	−1.268^c^	0.205	36.7 (36.5, 37.0)	36.7 (36.5, 36.8)
Heart rate, median[IQR], (times/min)	77 (68, 84)	79 (71, 88)	−1.669^c^	0.095	77 (70, 85)	79 (75, 82)
Respiratory frequency, median[IQR], (times/min)	20 (19, 20)	20 (20, 21)	−3.975^c^	**<0.001**	20 (19, 20)	20 (19, 20)
MAP, mean ± SD, (mmHg)	101 ± 15	104 ± 16	−1.641^a^	0.102	108 ± 15	110 ± 24
**GCS, *n* (%)**			41.959^b^	**<0.001**		
3–8	9 (4.9)	23 (30.3)			2 (2.1)	13 (39.4)
9–12	6 (3.3)	9 (11.8)			8 (8.4)	8 (24.2)
13–15	167 (91.8)	44 (57.9)			85 (89.5)	12 (36.4)
**WFNS, *n* (%)**			41.266^b^	**<0.001**		
I-III	167 (91.8)	44 (57.9)			85 (89.5)	12 (36.4)
VI-V	15 (8.2)	32 (42.1)			10 (10.5)	21 (63.6)
**mFisher, *n* (%)**			35.176^b^	**<0.001**		
0–2	155 (85.2)	38 (50.0)			74 (77.9)	7 (21.2)
3–4	27 (14.8)	38 (50.0)			21 (22.1)	26 (78.8)
**Hunt-Hess, *n* (%)**			33.883^b^	**<0.001**		
I–III	176 (96.7)	55 (72.4)			93 (97.9)	18 (54.5)
VI–V	6 (3.3)	21 (27.6)			2 (2.1)	15 (45.5)

^a^ is the *t*-value, ^b^ is the chi-square value, and ^c^ is the Z-value. The bold values mean the results of the univariate analysis in the modeling queue are statistically significant.

**Table 2:** First laboratory indicator information of the aSAH study cohort.

Variables	Modeling cohort (*N* = 258)				Validation cohort (*N* = 128)	
	Favorable outcome (*n* = 182)	Unfavorable outcome (*n* = 76)	Value	*p*	Favorable outcome (*n* = 95)	Unfavorable outcome (*n* = 33)
WBC, median[IQR], (×10^9^/L)	9.14 (6.93, 11.21)	11.02 (9.24, 13.70)	−5.003^c^	**<0.001**	9.90 (8.20, 11.60)	12.50 (10.50, 16.60)
NEUT, median[IQR], (×10^9^/L)	7.22 (5.30, 9.11)	9.67 (7.42, 12.54)	−5.803^c^	**<0.001**	8.00 (6.50, 9.58)	10.70 (9.06, 14.95)
LYM, median[IQR], (×10^9^/L)	1.20 (0.90, 1.57)	0.95 (0.64, 1.38)	−3.123^c^	**0.002**	1.20 (0.90, 1.60)	0.70 (0.55, 1.15)
MONO, median[IQR], (×10^9^/L)	0.49 (0.35, 0.65)	0.58 (0.41, 0.79)	−2.924^c^	**0.003**	0.51 (0.37, 0.65)	0.67 (0.51, 0.90)
RBC, median[IQR], (×10^12^/L)	4.06 (3.76, 4.51)	3.95 (3.64, 4.43)	−1.669^c^	0.095	4.13 (3.77, 4.41)	4.03 (3.43, 4.45)
Hb, median[IQR], (g/L)	120 (113, 133)	120 (106, 128)	−1.659^c^	0.097	127 (113, 136)	122 (105, 141)
PLT, median[IQR], (×10^9^/L)	199.0 (162.0, 225.7)	184.5 (159.5, 237.5)	−0.567^c^	0.570	215.0 (186.0, 250.0)	182.0 (141.0, 238.0)
ALB, median[IQR], (g/L)	43.00 (39.30, 45.00)	41.00 (38.25, 44.19)	−1.500^c^	0.134	43.00 (41.00, 46.00)	43.00 (39.00, 46.00)
Glu, median[IQR], (mmol/L)	6.83 (5.84, 8.30)	7.57 (6.18, 9.07)	−2.235^c^	0.025	6.73 (5.82, 8.42)	8.00 (7.05, 9.38)
CK, median[IQR], (U/L)	82.50 (48.10, 149.75)	98.17 (65.00, 229.25)	−2.279^c^	0.023	84.00 (56.00, 130.00)	125.00 (70.50, 243.51)
CK-MB, median[IQR], (U/L)	12.45 (9.00, 17.03)	14.00 (9.90, 19.10)	−1.757^c^	0.079	15.00 (11.00, 20.00)	19.10 (15.50, 27.50)
K+, mean ± SD, (mmol/L)	3.78 ± 0.40	3.70 ± 0.57	1.183^a^	0.239	3.77 ± 0.50	3.79 ± 0.54
Na+, mean ± SD, (mmol/L)	138.62 ± 4.09	138.98 ± 5.50	−0.515^a^	0.607	139.05 ± 3.26	139.96 ± 4.91
COP, mean ± SD, (mmol/L)	297.02 ± 9.01	298.38 ± 12.24	−0.862^a^	0.391	296.60 ± 7.26	301.98 ± 12.46
PT, median[IQR], (sec)	11.80 (10.88, 12.90)	11.40 (10.60, 12.70)	−1.679^c^	0.093	11.10 (10.60, 11.70)	11.10 (10.70, 11.95)
APTT, median[IQR], (sec)	26.90 (22.80, 33.00)	25.75 (23.25, 32.23)	−0.699^c^	0.484	24.90 (23.60, 26.90)	25.40 (23.15, 27.05)
Fib, median[IQR], (g/L)	2.88 (2.41, 3.61)	3.13 (2.66, 3.96)	−2.116^c^	0.034	2.83 (2.29, 3.39)	2.61 (2.46, 3.17)
D-dimer, median[IQR], (mg/L)	1.27 (0.71, 3.43)	2.80 (1.51, 5.49)	−4.005^c^	**<0.001**	1.14 (0.52, 2.43)	2.93 (1.77, 5.23)

^a^ is the *t*-value, ^b^ is the chi-square value, and ^c^ is the Z-value. The bold values mean the results of the univariate analysis in the modeling queue are statistically significant.

**Table 3:** Data on the aSAH study cohort during hospitalization.

Variables	Modeling cohort (*N* = 258)				Validation cohort (*N* = 128)	
	Favorable outcome *n* = 182	Unfavorable outcome *n* = 76	Value	*p*	Favorable outcome *n* = 95	Unfavorable outcome *n* = 33
LOS, median[IQR], (days)	16 (12, 19)	17 (11, 22)	−1.353^c^	0.176	15 (11, 18)	25 (11, 38)
MV duration, median[IQR], (days)	0 (0, 0)	0 (0, 3.8)	−6.712^c^	**<0.001**	0 (0, 0)	3 (0, 9)
Aneurysm size, median[IQR], (mm)	5.2 (4.0, 7.0)	5.8 (3.8, 7.0)	−0.214^c^	0.830	5.3 (4.0, 7.3)	6.7 (4.7, 8.0)
**Aneurysm location, *n* (%)**			5.475^b^	0.140		
Anterior circulation	67 (36.8)	37 (48.7)			37 (38.9)	15 (45.5)
Internal carotid artery	81 (44.5)	25 (32.9)			38 (40.0)	13 (39.4)
Middle cerebral artery	17 (9.3)	4 (5.3)			11 (11.6)	2 (6.1)
Posterior circulation	17 (9.3)	10 (13.2)			9 (9.5)	3 (9.1)
Number of aneurysms, median[IQR]	1 (1, 1)	1 (1, 2)	−0.583^c^	0.560	1 (1, 1)	1 (1, 1)
**Timing of interventional surgery, *n* (%)**			2.661^b^	0.264		
0–3d	117 (64.3)	56 (73.7)			74 (77.9)	28 (84.8)
3–10d	50 (27.5)	17 (22.4)			17 (17.9)	4 (12.1)
>10d	15 (8.2)	3 (3.9)			4 (4.2)	1 (3.0)
**Drainage methods, *n* (%)**			16.298^b^	**<0.001**		
None	92 (50.5)	29 (38.2)			33 (34.7)	5 (15.2)
One type	88 (48.4)	38 (50.0)			60 (63.2)	24 (72.7)
Two or more types	2 (1.1)	9 (11.8)			2 (2.1)	4 (12.1)
Secondary cerebral infarction, *n* (%)	20 (11.0)	40 (52.6)	52.091^b^	**<0.001**	10 (10.5)	15 (45.5)
Rebleeding, *n* (%)	7 (3.8)	5 (6.6)	–	0.344	4 (4.2)	4 (12.1)
Pulmonary infection, *n* (%)	65 (35.7)	65 (85.5)	53.213^b^	**<0.001**	52 (54.7)	29 (87.9)
Hydrocephalus, *n* (%)	18 (9.9)	20 (26.3)	11.517^b^	**0.001**	12 (12.6)	10 (30.3)
Epilepsy, *n* (%)	13 (7.1)	11 (14.5)	3.415^b^	0.065	3 (3.2)	4 (12.1)
Cerebral hernia, *n* (%)	4 (2.2)	12 (15.8)	17.026^b^	**<0.001**	1 (1.1)	10 (30.3)
Cardiac event, *n* (%)	20 (11.0)	25 (32.9)	17.866^b^	**<0.001**	9 (9.5)	16 (48.5)

^a^ is the *t*-value, ^b^ is the chi-square value, and ^c^ is the Z-value. The bold values mean the results of the univariate analysis in the modeling queue are statistically significant.

Multicollinearity tests revealed high multicollinearity between GCS (Tol 0.040, VIF 24.824) and WFNS (Tol 0.062, VIF 16.195), as well as between the first white blood cell count (Tol 0.029, VIF 34.450) and the first neutrophil count on admission (Tol 0.032, VIF 30.845). To address this multicollinearity issue, GCS and the first neutrophil count on admission were excluded. Instead, WFNS and the first leukocyte counts on admission, along with other clinically significant variables identified through univariate analyses, were included in the multivariable logistic regression analysis. The multivariable backward stepwise logistic regression analysis showed that age (*p* < 0.001, OR = 1.085, 95%CI = 1.044–1.128), WFNS grade of IV-V (*p* = 0.018, OR = 3.746, 95%CI = 1.248–11.241), mFisher grade of 3–4 (*p* = 0.018, OR = 2.903, 95%CI = 1.198–7.035), secondary brain infarction (*p* < 0.001, OR = 12.966, 95%CI = 5.218–32.222), and the first leukocyte counts on admission (*p* < 0.001, OR = 1.326, 95%CI = 1.153–1.525) were identified as independent risk factors of a 6-month adverse outcome (Table 4).

**Table 4:** Multivariate logistic regression analysis of poor prognosis for aSAH patients in the modeling cohort.

Variables	β	SE	*p*	OR (95%CI)
Age	0.082	0.020	<0.001	1.085 (1.044–1.128)
WFNS IV-V	1.321	0.561	0.018	3.746 (1.248–11.241)
mFisher 3–4	1.066	0.452	0.018	2.903 (1.198–7.035)
Secondary cerebral infarction	2.562	0.464	<0.001	12.966 (5.218–32.222)
Leukocyte counts on admission	0.282	0.071	<0.001	1.326 (1.153–1.525)
**Goodness-of-fit test**				
X^2^				9.176
Degrees of freedom				8
*p*				0.328

### Construction of nomogram

**Figure 1:**
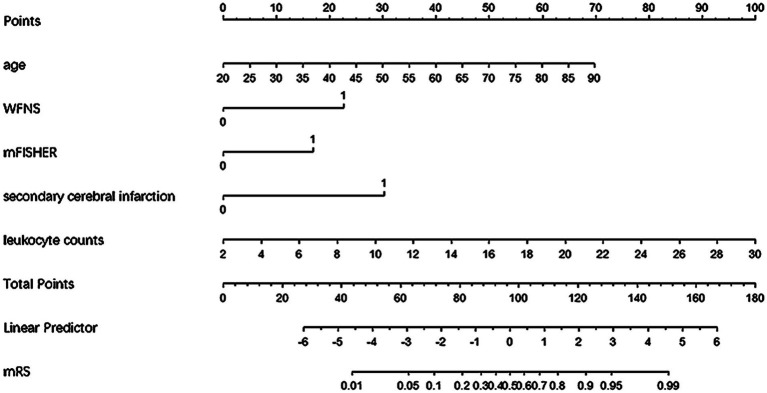
Nomogram for predicting poor prognosis in aSAH patients undergoing interventional embolization.

We combined independent risk predictors of 6-month adverse outcomes into a logistic regression model and ultimately constructed a nomogram to predict the risk of unfavorable outcomes at 6 months for patients with aSAH undergoing interventional embolization (Figure 1).

### Validation of nomogram

#### Discrimination evaluation

**Figure 2:**
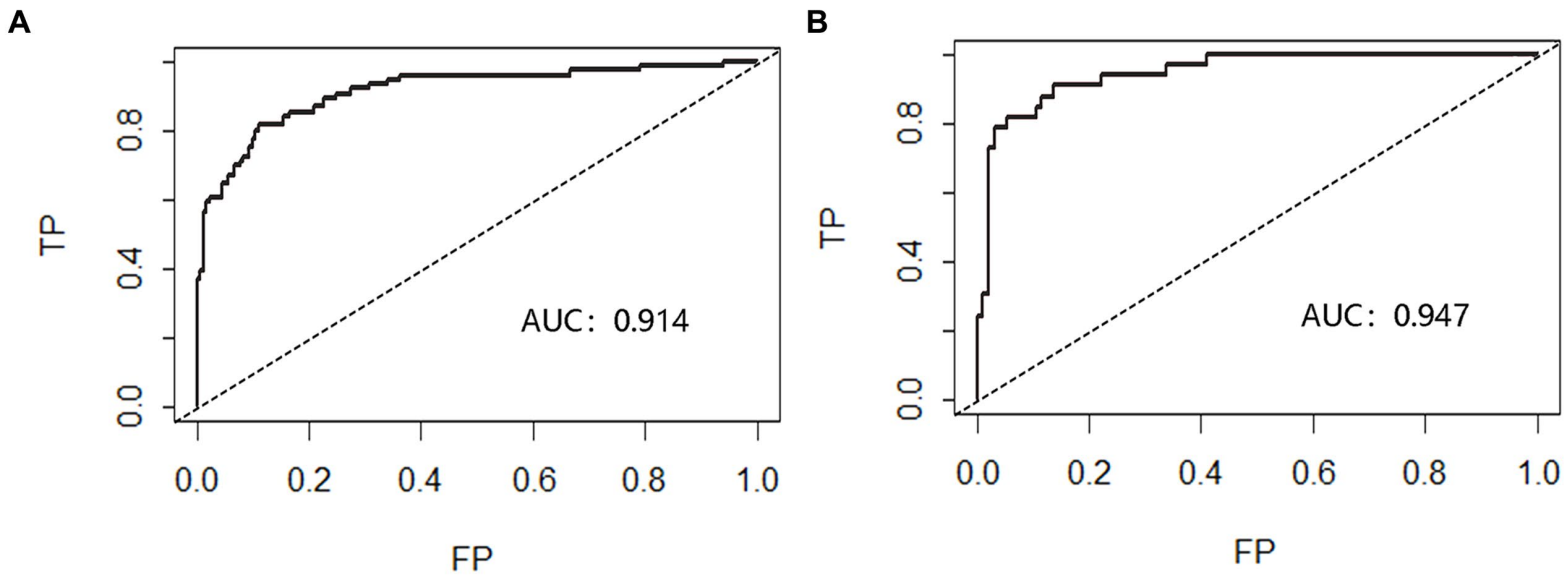
Receiver operating characteristic curve (ROC) validation of nomogram in aSAH patients. The risk prediction’s true positive rate is represented by the Y-axis, while its false positive rate is represented by the X-axis. The nomogram’s performance in the modeling queue **(A)** and validation queue **(B)** is shown by the black curve.

The nomogram’s discrimination ability was assessed using the ROC curve, with an AUC of 0.914 (*p* < 0.001, 95%CI = 0.873–0.956) for the predictive model in the modeling cohort and 0.947 (*p* < 0.001, 95%CI = 0.907–0.987) for the predictive model in the validation cohort (Figure 2).

#### Calibration evaluation

**Figure 3:**
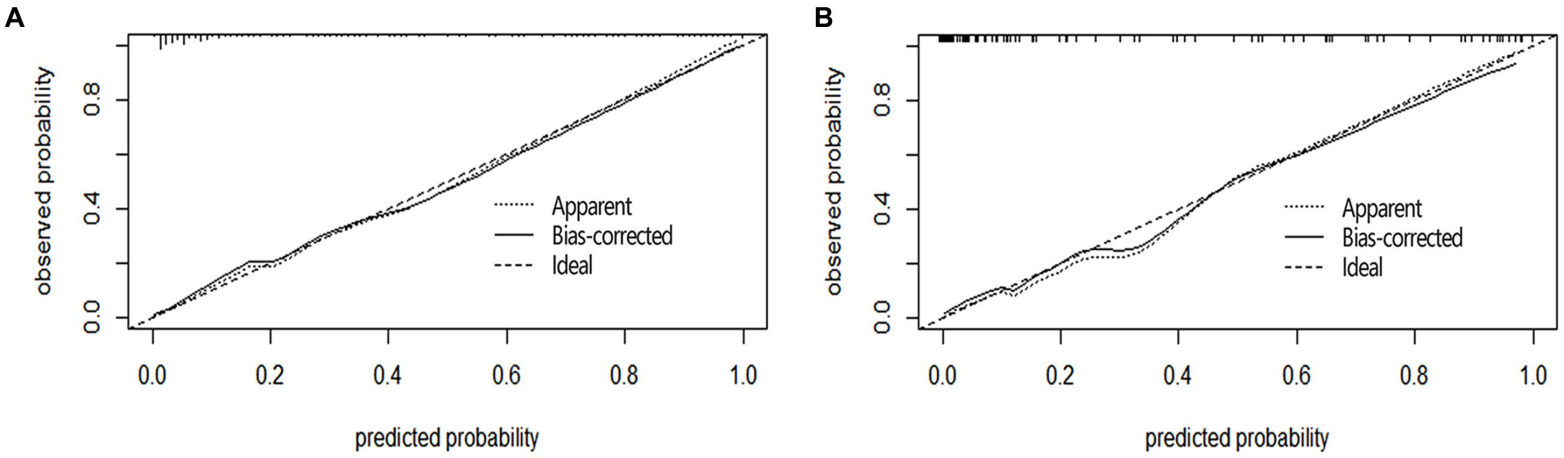
Calibration curves of nomogram in aSAH patients. The Y-axis represents the actual probability of poor prognosis in the aSAH patients, and the X-axis represents the predicted probability of poor prognosis in the aSAH patients. The diagonal dashed line, characterized by a slope of 1, symbolizes a flawless prediction from an ideal model. Meanwhile, the solid line depicts the prediction model’s performance in both the modeling queue **(A)** and the validation queue **(B)**. The results show that the closer to the diagonal dashed line, the better the prediction.

Calibration curve plots and the Hosmer-Lemeshow goodness-of-fit test were used to assess the calibration of the nomogram. The Brier score for the modeling cohort was 0.097, and the Brier score for the validation cohort was 0.078. Additionally, both calibration curves showed a high degree of overlap with the standard curve, indicating good consistency between the clinical prediction model and the actual outcomes (Figure 3). The Hosmer-Lemeshow goodness-of-fit test was also performed, with the results showing X^2^ = 9.176 and *p* = 0.328 (>0.05) for the modeling cohort, and X^2^ = 11.348 and *p* = 0.183 (>0.05) for the validation cohort.

### Evaluation of clinical applicability

**Figure 4:**
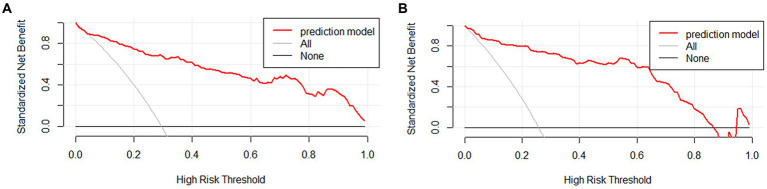
Clinical decision curves of nomogram in aSAH patients. The Y-axis illustrates the net benefit derived from the undertaken action, while the X-axis portrays the risk associated with an unfavorable prognosis. The slanted thin gray line represents the assumption that all patients take the intervention, the horizontal thick solid line represents that no patient takes the intervention, and the aSAH nomogram is depicted by the red curve in both the modeling queue **(A)** and the validation queue **(B)**.

**Figure 5:**
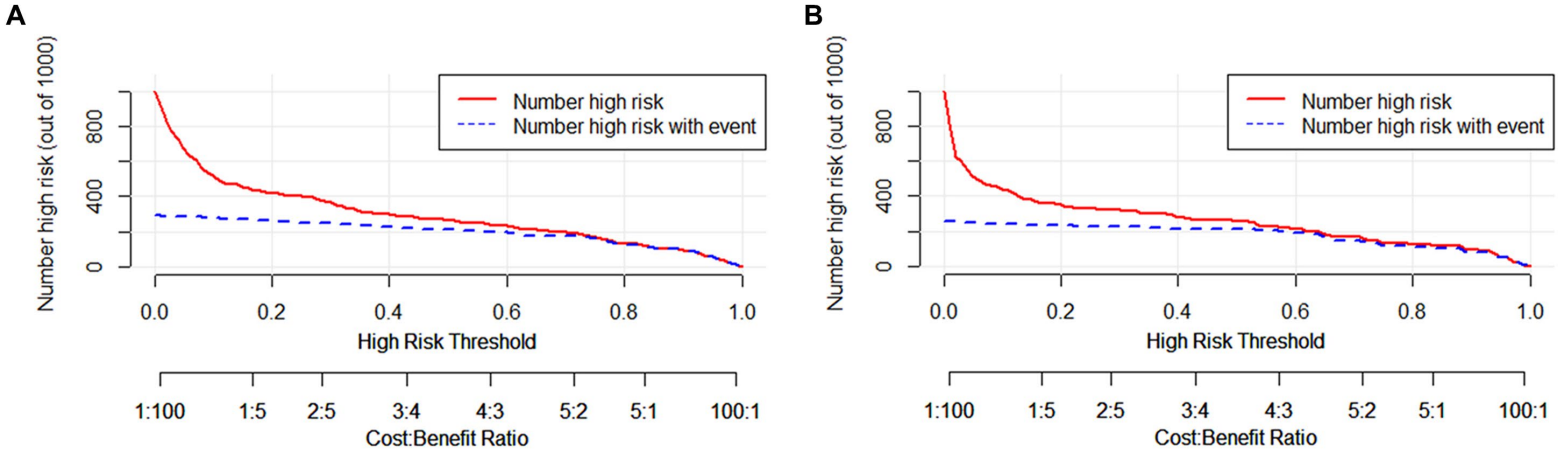
Clinical impact curves of nomogram in aSAH patients. The number of individuals the model identified as positive (high risk of poor prognosis) at each threshold probability is shown by the solid line, while the number of real positives at each threshold probability is shown by the dotted line. **(A)** From the modeling cohort, and **(B)** from the validation cohort.

The clinical applicability of the nomogram was assessed using clinical decision curve analysis (DCA) and clinical impact curve (CIC). From the DCA, in the modeling cohort, the nomogram used to predict the risk of adverse outcomes showed greater net benefits when the threshold probability ranged from 6 to 100% compared to all patients or none of them treated with intervention embolization. In the validation cohort, the nomogram showed greater net benefits when the threshold probability was between 2 to 88% and 95 to 100% compared to all patients or none of them treated with intervention embolization (Figure 4). From the CIC, when the threshold probability was >20%, the nomogram categorized several individuals as “positive” (high risk) in close concordance with the count of true positives, both within the modeling cohort and the validation cohort (Figure 5).

### Evaluation of generalizability

The generalizability of the nomogram was assessed using the bootstrap method, to avoid underfitting or overfitting of the nomogram. The findings indicated that it demonstrated good accuracy and stability in predicting adverse outcomes (Accuracy = 0.861, Kappa = 0.646).

### Comparison with other aSAH prognostic scoring systems and its relevant variables

**Figure 6:**
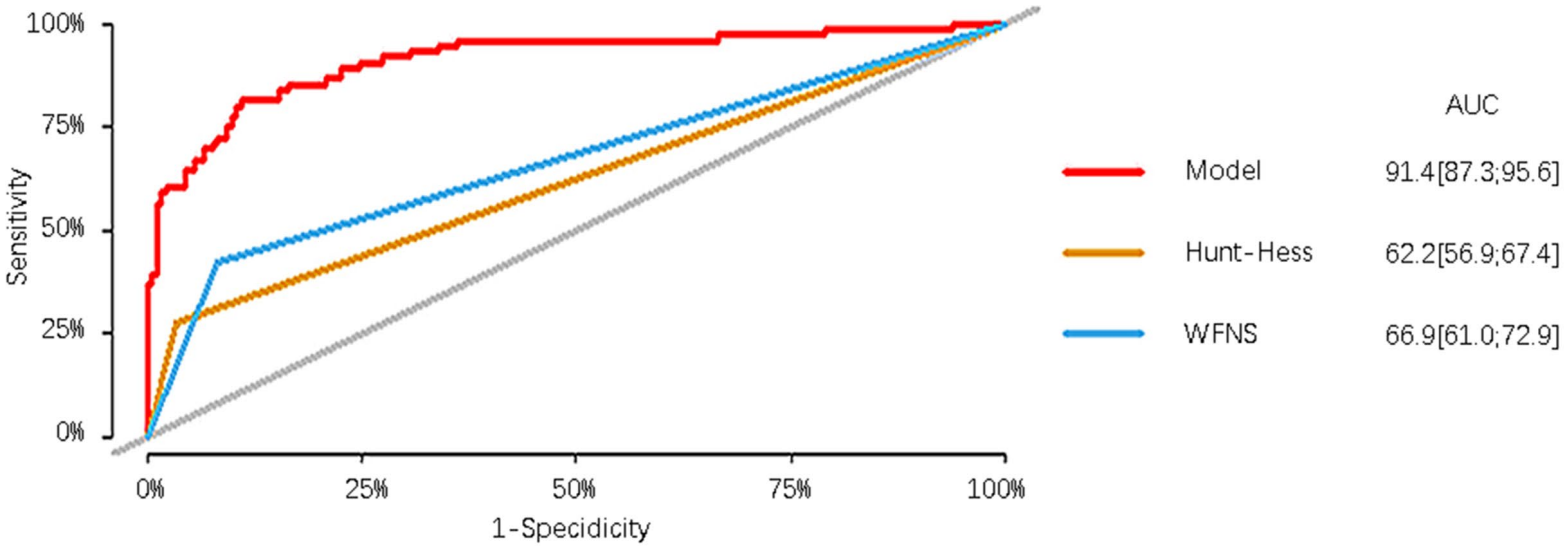
Comparison of the nomogram with WFNS and Hunt-Hess.

In the modeling cohort, the nomogram derived from the study was compared with traditional and well-established aSAH prognostic scoring systems. The nomogram showed a significantly higher AUC of 0.914 (95%CI = 0.873–0.956) compared to the Hunt-Hess grading system with an AUC of 0.622 (95%CI = 0.569–0.674) and the WFNS grading system with an AUC of 0.669 (95%CI = 0.610–0.729). These differences were statistically significant (Table 5; Figure 6).

**Table 5:** Comparison of the nomogram with WFNS and Hunt-Hess in the modeling group.

Comparison of different scales			Z/D	*p*
Nomogram	vs.	WFNS	6.618	<0.001
Nomogram	vs.	Hunt-Hess	8.577	<0.001
WFNS	vs.	Hunt-Hess	−2.180	0.029

**Figure 7:**
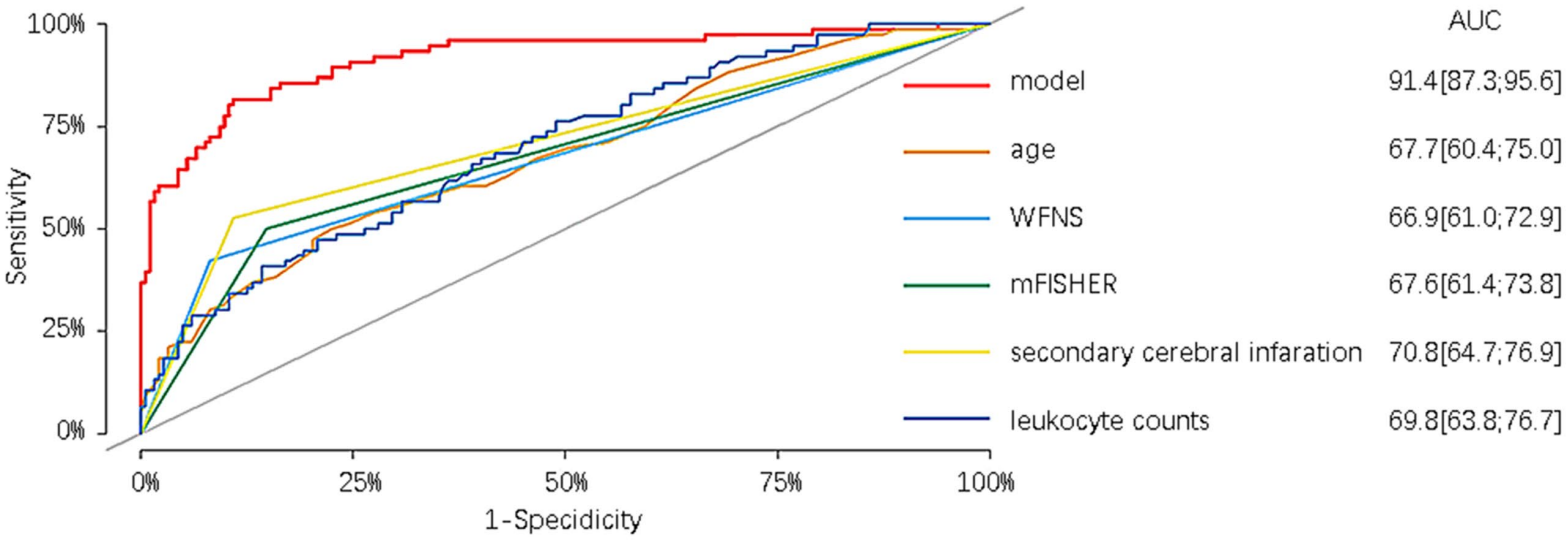
Comparison of the nomogram with its component variables.

Furthermore, in the modeling cohort, the nomogram was compared with its component variables. The nomogram demonstrated a greater AUC of 0.914 (95%CI = 0.873–0.956) compared to age with an AUC of 0.677 (95%CI = 0.604–0.750), WFNS with an AUC of 0.669 (95%CI = 0.610–0.729), mFisher with an AUC of 0.676 (95%CI = 0.614–0.738), secondary brain infarction with an AUC of 0.708 (95%CI = 0.647–0.769), and initial leukocyte counts on admission with an AUC of 0.698 (95%CI = 0.628–0.767) (Figure 7). We further compare the created models with the previously presented SAHIT, SAFIRE, and Fresh models, the results of which we show in the Supplementary material.

## Discussion

With the continuous development of neurointerventional techniques and surgical materials, the clinical outcomes associated with endovascular treatment for aSAH have garnered increasing attention. A pivotal large-scale randomized clinical trial, the International Subarachnoid Aneurysm Trial (ISAT) (26), unequivocally demonstrated the superior clinical efficacy of endovascular coiling over traditional microsurgical clipping in the treatment of ruptured intracranial aneurysms. This therapeutic approach is increasingly gaining widespread adoption as a prominent tool for managing patients with aSAH, particularly among elderly individuals. Over recent years, neurosurgeons, both at the domestic and international levels, have endeavored to develop diverse prognostic models for subarachnoid hemorrhage, incorporating various influential factors. However, the majority of these models are hybrids, capable of predicting outcomes for both interventional embolization and clipping therapies. Models exclusively tailored for embolization are relatively scarce, and each of them exhibits distinct limitations. Therefore, our research has developed a visual predictive model aimed at estimating the likelihood of adverse outcomes among aSAH patients 6 months following interventional embolization therapy. This model encompasses demographic data, clinical symptoms and admission consciousness scores, initial imaging assessments, in-hospital complications, and the first admission laboratory analyses. These variables are routinely encountered in clinical practice and easily accessible, rendering the model highly practical. The model’s discrimination, calibration, and clinical utility have all been robustly validated, with satisfactory results from internal validation.

We also compared the derived nomogram with two well-established prognostic scoring systems widely employed in clinical practice, namely the WFNS and Hunt-Hess grading scales. Our findings revealed the AUC values of our nomogram were significantly higher than those of the two scoring systems, indicating that our nomogram can relatively accurately predict the prognosis of aSAH patients undergoing interventional embolization. The classical scoring scales showed poor predictive efficacy in our research data. We speculate that although the two classic scoring systems have been used for many years in the assessment of patient prognosis, different doctors may assign different GCS scores to the same patient, potentially leading to a certain degree of error. Moreover, the two scales have a limited role in predicting prognosis based only on clinical manifestations at admission, ignoring serious complications such as rebleeding, secondary cerebral infarction, cerebral herniation, and circulatory failure that may occur during the patient’s hospitalization. In our modeling cohort of 76 patients with poor prognosis, 55 (72.3%) patients were admitted with a good Hunt-Hess classification, of whom 52 (94.5%) were comorbid with one or more complications, and 44 (57.9%) patients were admitted with a good WFNS classification, of whom 41 (93.2%) were comorbid with one or more complications. Similar results were observed in the validation cohort. To exemplify the strengths of our new prediction model, in addition to comparing it with other external rating scales, we also compared the prediction model with its constituent variables. The results reveal that the prediction performance of our model surpasses that of age, WFNS classification, mFisher score, secondary cerebral infarction, and the first white blood cell count upon admission.

Our study established age as an independent risk factor associated with unfavorable prognoses at the 6-month post-endovascular treatment in patients diagnosed with aSAH. Adverse outcomes were more common in the elderly cohort (age ≥ 60 years). Despite the many advantages of endovascular intervention, which has become an important treatment method for aSAH patients, older patients often experience higher perioperative complications, including subarachnoid clot formation and detachment, hydrocephalus, higher rebleeding rates, as well as pre-existing comorbidities such as diabetes, hypertension, and cardiopulmonary diseases (27). Although the definition of elderly individuals in the field of aSAH may vary among published articles, it is consistent with the outcomes of the International Subarachnoid Aneurysm Trial (26) that age is an inverse predictor of prognosis.

The current WFNS grading scale, introduced in 1988, is widely used for grading the severity and predicting the prognosis of aSAH. In our study, we also used the widely accepted WFNS grading scale. We categorized the WFNS grades into I-III and IV-V, aiming to avoid subtle distinctions between different grades while maximizing the differentiation between favorable and unfavorable outcomes in aSAH patients. The multivariate analysis also indicated that Patients with WFNS grades IV-V upon admission had a significantly increased chance of experiencing a poor outcome after 6 months following aSAH. This is consistent with previous research findings. Shen et al. (28) collected and analyzed data from 147 aSAH patients with poor prognosis, and their results suggested that WFNS grade V was an important predictive factor for poor prognosis. Furthermore, Li et al. (29) proposed a novel scoring model named “TAPS”, utilizing Early Brain Injury (EBI) markers to forecast the functional outcomes of patients with aSAH at the 90-day time point. This model also included WFNS grades IV-V. Schuss et al. (30) also have affirmed that WFNS grade V stands out as a robust predictor of an adverse prognosis among individuals with aSAH. In general, the WFNS classification can better respond to the neurological impairment of patients at the time of admission, indicating that the severity of neurological impairment in the early stage can determine the prognosis of patients to a certain extent, which is important for assessing the degree of risk of aSAH.

In our study, we also utilized the modified Fisher scoring system to assess the amount of subarachnoid hemorrhage. The modified Fisher score, introduced in 2006, emphasizes the presence of intraventricular hemorrhage (IVH) and the thickness of blood in the cisterns. Compared to the original Fisher score, the modified version provides a more accurate prediction of symptomatic vasospasm after aSAH and demonstrates superior in predicting the occurrence of new cerebral infarctions and patient outcomes. A study (13) based on a cohort of 271 aSAH patients aimed to analyze the relationship between the increase in various scores (Fisher, modified Fisher, and Claassen scale) assessed from patients’ admission CT scans and the risk of subsequent complications. The results revealed that, compared to Fisher and Claassen scales, the relationship between an increase in the modified Fisher score and the incidence of complications was more linear. Within this scoring system, each additional point was linked to a heightened likelihood of experiencing vasospasm, delayed cerebral infarction, and an unfavorable prognosis. The study conducted by Oliveira et al. (14) also discovered that the Fisher Revised Scale (FRS) may be more effective in identifying patients at risk of clinical vasospasm and neurological deterioration. Similarly, in our study, the modeling cohort had 25.2% of patients with severe imaging manifestations (mFisher score 3–4), of which 58.5% had a poor prognosis. The heavier the patient’s imaging score, the worse the prognosis, and the difference was statistically significant (*p* < 0.001). The potential explanation lies in the sudden surge of intracranial pressure after the rupture of an arterial aneurysm. This event triggers a decline in cerebral perfusion pressure and compromises the autoregulatory function, which may ultimately lead to transient or persistent ischemia. The volume of blood entering the subarachnoid space correlates with the peak ICP at the moment of aneurysm rupture, according to the Monro-Kellie doctrine. The substantial correlations between the imaging results of individuals with aSAH and the initial clinical presentation and prognosis can be explained by this relationship between the burden of intracranial hemorrhage and the severity of early brain injury (31, 32). However, it is worth noting that relying solely on clinical grading or radiological scoring to assess the prognosis of aSAH may result in significant deviations in predicting patient outcomes.

There is no doubt that post morbidity complications have a significant impact on the adverse prognosis of individuals with aSAH. Our study collected relevant data on post-illness complications such as cerebral infarction, rebleeding, lung infection, hydrocephalus, seizures, brain herniation, and cardiovascular events for all included patients. The final results also demonstrated fair predictive efficacy of secondary cerebral infarction on the 6-month prognosis of patients undergoing interventional embolization therapy, with an AUC value of 0.708 (95%CI = 0.647–0.769). The reasons for this may be related to the initiation of cell death mechanisms, disruption of the blood–brain barrier, and acute inflammatory responses during the acute phase of SAH. All of these factors could contribute to the occurrence of brain edema, which itself is a factor influencing prognosis. Additionally, acute hemodynamic instability may lead to microvascular spasm, microthrombus formation, and failure of cerebral autoregulation. All of these factors may be involved in the sustained ischemic injury following SAH and result in delayed manifestations (33). The impact of cerebral infarction on the prognosis of SAH is supported by several key findings. Vergouwen et al. (34) suggested that independent of angiographic vasospasm, cerebral infarction has a direct influence on prognosis, making it an important research target for improving SAH outcomes. Taki et al. (35) reported that factors including cerebral infarction induced by both endovascular coiling and vasospasm significantly affect adverse outcomes. Kanamaru et al. (36) demonstrated that cerebral infarction, regardless of its underlying cause, significantly influences the poor prognosis after SAH, and cerebral vasospasm remains the most crucial potential breakthrough for treatment. In a study by Su et al. (37), they also found that the short-term prognosis of aSAH hospitalized patients in the cerebral infarction group was significantly worse than that of the non-cerebral infarction group, and that the patients’ degree of cognitive and sensorymotor impairment usually determines the length of hospitalization, which could explain why the average length of hospitalization in the group of cerebral infarcted patients was longer and the outcome was poorer.

In our study, secondary cerebral infarction is not directly equivalent to aneurysm embolization itself, but rather associated with potential complications (such as vasospasm or delayed cerebral ischemia) that may occur after the onset of the disease. Although our study did not further differentiate the specific cases of cerebral infarction after subarachnoid hemorrhage. However, regardless of the mechanism, cerebral infarction is the ultimate manifestation of various pathways of neurologic injury, all of which significantly contribute to the poor prognosis of SAH. In addition most studies tend to exclude cerebral infarctions due to medical factors or medical decision-making factors, which equally have an impact on prognostic prediction. The data from our study, which included all cases of cerebral infarction occurring after aSAH, confirmed that there was a notable disparity in secondary cerebral infarction rates between the groups with good and poor prognosis. It has been recognized as a distinct risk factor independently associated with an adverse prognosis at the 6-month follow-up in patients undergoing interventional treatments. The prediction model we constructed included relevant complication variables, which may help improve the sensitivity in predicting adverse events.

Our nomogram also emphasizes the importance of preoperative inflammatory biomarkers, further highlighting the significance of neurogenic inflammatory response during the onset of aSAH. This could be because uncontrolled inflammation occurs after early brain injury as a result of extravascular blood response, decreased brain autoregulation, product release from injured brain tissue, and ischemia–reperfusion injury. Extracellular vesicles formed from astrocytes are released in response to pro-inflammatory cytokines like interleukin-1β (IL-1β). These vesicles penetrate the peripheral circulation and facilitate leukocyte migration towards the central nervous system. When peripheral leukocytes move into the brain and cerebrospinal fluid, active neutrophils cause damage to cerebral microvessels, worsening the effects of ischemia injury. The severe inflammatory response within the system that follows SAH peaks in 24 to 48 h and causes a delay in the deterioration of the nervous system (38).

Studies by Mahta et al. (39), Muroi et al. (40), and Srinivasan et al. (41) have all demonstrated that the early inflammatory response occupies a significant position in the pathophysiology of SAH. In our study, there appeared to be a mild elevation in the white blood cell count upon admission, which is a relatively common finding in the early stages following SAH. We also observed that leukocyte counts tended to be higher in patients with aSAH who had a poor prognosis at 6 months, and the observed elevation in leukocyte counts reached statistical significance. While the high proportion of infectious complications in patients with poor prognosis may be an expected fact influencing white blood cell count, our study included patients within 72 h of the onset of illness, and the elevation in leukocyte counts at admission monitoring mostly preceded the occurrence of infectious complications.

Furthermore, another negative consequence of the inflammatory response following SAH may be the impairment of organ functions, which can have varying degrees of impact on prognosis. Researchers have observed that the systemic inflammatory response after aSAH may play a role in cardiac dysfunction, as their studies have revealed an independent correlation between elevated total leukocytes and neutrophils and cardiac injury in aSAH patients (42). Likewise, the occurrence of systemic inflammatory response syndrome might result in the progression of acute lung injury (43). In the practical work of clinical physicians, the blood routine examination in aSAH patients upon admission may serve as a simple method to help assess the potential for an adverse prognosis in patients.

This study has several limitations, firstly, we used a retrospective collection of statistical data. Secondly, when assessing functional neurological outcomes, we intentionally set the follow-up point at 6 months post-discharge, recognizing it as a crucial period for neurological recovery. However, it would have been more accurate to obtain data for long-term follow-up. In addition, although the study data were obtained from different centers, the two centers were closely related, with varying degrees of sharing in terms of medical technology, staffing, and means of patient care, which may have led to unavoidable bias in the analyses and conclusions. Further external validation of the nomogram in larger multicentre studies is required.

## Conclusion

The nomogram we constructed includes five variables: age, WFNS grades of IV-V, mFisher score of 3–4, secondary cerebral infarction and the first leukocyte counts on admission, which can early and effectively predict the 6-month prognosis of patients with aSAH who underwent interventional embolization, with good differentiation, calibration and clinical applicability. The model allows for a relatively accurate assessment of patients’ prognosis in the early stages of the disease, providing reliable evidence-based medicine to assist doctors in taking necessary intervention measures for high-risk patients as early as possible.

## Data Availability

The original contributions presented in the study are included in the article/Supplementary material, further inquiries can be directed to the corresponding author.
