# Supplementary material for: Innovative prognostication: a novel nomogram for post-interventional aneurysmal subarachnoid hemorrhage patients
Source: Front Neurol. 2024 Aug 19;15:1410735. doi: 10.3389/fneur.2024.1410735 (PMC11369945; doi:10.3389/fneur.2024.1410735)
Supplement: Supplementary file 1 [file Image_1.pdf]

# Supplementary Figure

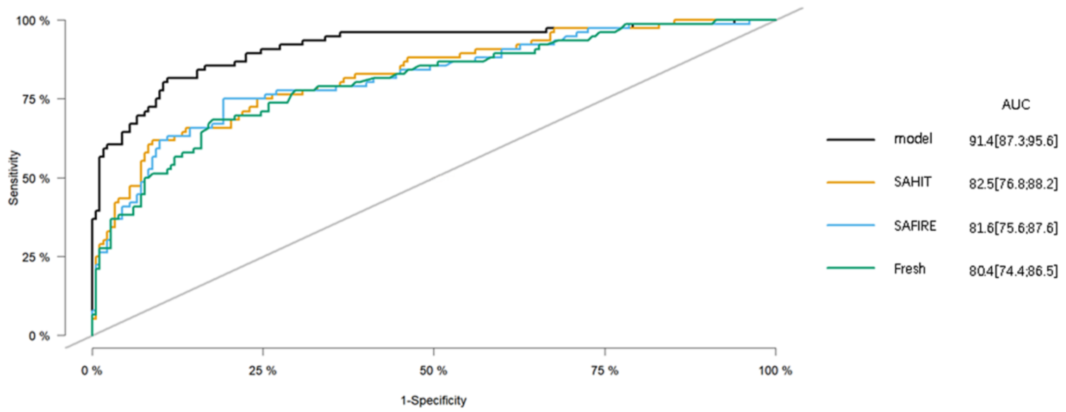


Comparison of the AUCs between our models and those of SAHIT, SAFIRE, and Fresh in the modeling cohort.
